# Presence of Chromosomal *crpP*-*like* Genes Is Not Always Associated with Ciprofloxacin Resistance in *Pseudomonas aeruginosa* Clinical Isolates Recovered in ICU Patients from Portugal and Spain

**DOI:** 10.3390/microorganisms9020388

**Published:** 2021-02-14

**Authors:** Marta Hernández-García, María García-Castillo, Sergio García-Fernández, Diego López-Mendoza, Jazmín Díaz-Regañón, João Romano, Leonor Pássaro, Laura Paixão, Rafael Cantón

**Affiliations:** 1 Servicio de Microbiología, Hospital Universitario Ramón y Cajal and Instituto Ramón y Cajal de Investigación Sanitaria (IRYCIS), 28034 Madrid, Spain; merygarciac@gmail.com (M.G.-C.); segarciafe@hotmail.com (S.G.-F.); rafael.canton@salud.madrid.org (R.C.); 2 Red Española de Investigación en Patología Infecciosa (REIPI), 28029 Madrid, Spain; 3 Departamento Médico, MSD España, 28027 Madrid, Spain; diego.lopez@merck.com (D.L.-M.); jazmin.diaz-reganon@merck.com (J.D.-R.); 4 MSD Portugal, 2770-192 Paço de Arcos, Portugal; joao.romano@merck.com (J.R.); leonor.passaro@merck.com (L.P.); laura.paixao@merck.com (L.P.)

**Keywords:** *Pseudomonas aeruginosa*, *crpP* gene, pathogenicity genomic islands, ciprofloxacin susceptibility, Spain, Portugal

## Abstract

CrpP enzymes have been recently described as a novel ciprofloxacin-resistance mechanism. We investigated by whole genome sequencing the presence of *crpP*-genes and other mechanisms involved in quinolone resistance in MDR/XDR-*Pseudomonas aeruginosa* isolates (*n* = 55) with both ceftolozane-tazobactam susceptible or resistant profiles recovered from intensive care unit patients during the STEP (Portugal) and SUPERIOR (Spain) surveillance studies. Ciprofloxacin resistance was associated with mutations in the *gyrA* and *parC* genes. Additionally, plasmid-mediated genes (*qnrS2* and *aac(6′)-Ib-cr*) were eventually detected. Ten chromosomal *crpP-like* genes contained in related pathogenicity genomic islands and 6 different CrpP (CrpP1-CrpP6) proteins were found in 65% (36/55) of the isolates. Dissemination of CrpP variants was observed among non-related clones of both countries, including the CC175 (Spain) high-risk clone and CC348 (Portugal) clone. Interestingly, 5 of 6 variants (CrpP1-CrpP5) carried missense mutations in an amino acid position (Gly7) previously defined as essential conferring ciprofloxacin resistance, and decreased ciprofloxacin susceptibility was only associated with the novel CrpP6 protein. In our collection, ciprofloxacin resistance was mainly due to chromosomal mutations in the *gyrA* and *parC* genes. However, *crpP* genes carrying mutations essential for protein function (G7, I26) and associated with a restored ciprofloxacin susceptibility were predominant. Despite the presence of *crpP* genes is not always associated with ciprofloxacin resistance, the risk of emergence of novel CrpP variants with a higher ability to affect quinolones is increasing. Furthermore, the spread of *crpP* genes in highly mobilizable genomic islands among related and non-related *P. aeruginosa* clones alert the dispersion of MDR pathogens in hospital settings.

## 1. Introduction

Quinolones are an important broad-spectrum antimicrobial group and represent one of the most frequently used classes of antibacterial drugs. Nevertheless, due to their increasing use, resistance to this group of antimicrobials has progressively being increased over the last thirty years, and quinolone resistance is included in the definition of multi-drug resistant (MDR) microorganisms and difficult to treat resistant (DTR) pathogens [1,2]. Resistance to quinolones usually results from mutational alterations in drug target affinity, efflux pumps and/or porin channels overexpression, and acquisition of resistance-conferring genes [3]. Chromosomal mutational resistance affecting fluroquinolones mainly occurs in the so-called quinolone resistance determining region (QRDR) of topoisomerases [4]. Moreover, plasmid-mediated quinolone resistance (PMQR) has been also described and is generally due to Qnr-type proteins, the aminoglycoside-modifying enzyme AAC(6′)-Ib-cr acetyltransferase and mobile efflux systems such as QepA or OqxAB [5]. Increasing attention to these plasmid-mediated resistance mechanisms has been given in the last year as they are commonly presented in extended spectrum beta-lactamase (ESBL) and carbapenemases producing microorganisms [6].

Ciprofloxacin is the quinolone most frequently used to treat infections caused by *Pseudomonas aeruginosa* either orally or intravenously [7]. Resistance to ciprofloxacin in this pathogen is mainly due to QRDR mutations, but PMQR has also been encountered [8]. CrpP is a recently described 65-amino-acid ciprofloxacin-modifying enzyme that confers decreased susceptibility to ciprofloxacin, norfloxacin and moxifloxacin, but not levofloxacin, by enzymatic phosphorylation [9]. *crpP* gene was first detected in 2018 as encoded within the pUM505 plasmid in a *P. aeruginosa* clinical isolate from Mexico [9]. Since then, ciprofloxacin-modifying *crpP* genes have been identified in *P. aeruginosa* as part of different mobile integrative and conjugative elements (ICEs) that frequently transfer horizontally [10]. However, homologous *crpP* genes have also been located in chromosomal pathogenicity genomic islands (PAGI) of *P. aeruginosa* clinical isolates recovered in France and Switzerland between 2000 and 2015 [11]. Additionally, a recent study has supported that mutations in the codons encoding Gly7, Asp9, Lys33 and Cys40 of CrpP protein generate modified CrpP variants that restore ciprofloxacin susceptibility [12].

According to recent literature, the novel crpP-*like* gene is widespread in *P. aeruginosa* clinical isolates, but it is also easily transferable to other bacterial species, including ESBL-producing *Enterobacterales*, contributing to the emergence of other multidrug resistance pathogens [13]. In Mexico, it was found in 5.2% of a collection of ESBL producing *Enterobacterales.* Nevertheless, the role of ciprofloxacin-modifying *crpP* genes and its prevalence among the hospital circulating *P. aeruginosa* isolates remain undetermined, particularly in hospital settings such as intensive care units (ICUs), where fluoroquinolones are heavily used. Our aim was to investigate the dissemination of *crpP*-*like* genes in MDR or extensively resistant (XDR) *P. aeruginosa* clinical isolates recovered in patients admitted in Spanish and Portuguese ICUs between 2016 and 2018. These isolates were obtained during surveillance studies to monitor the activity of ceftolozane-tazobactam, a novel β-lactam-β -lactamase combination with enhanced antimicrobial activity against *P. aeruginosa*, including MDR/XDR isolates [14,15,16].

## 2. Materials and Methods 

### 2.1. Pseudomonas aeruginosa Isolates and Antimicrobial Susceptibility

A total of 476 MDR/XDR *P. aeruginosa* isolates causing UTI (urinary tract infections), IAI (intra-abdominal infections) or LRTI (lower respiratory tract infections) were recovered from 11 Portuguese (*n* = 396) and 8 Spanish ICUs (*n* = 80) from different hospitals as a part of the STEP and SUPERIOR surveillance studies, respectively [14,15]. Minimum inhibitory concentration (MIC) values were determined by standard broth microdilution and interpreted according to the EUCAST 2020 criteria (https://www.eucast.org/fileadmin/src/media/PDFs/EUCAST_files/Breakpoint_tables/v_10.0_Breakpoint_Tables.pdf, accessed on 10 February 2021) (S, susceptible ≤ 0.001 mg/L; I, susceptible, increased exposure >0.001–0.5 mg/L; R, resistant > 0.5 mg/L) [14,15]. A subset of 55 *P. aeruginosa* isolates with susceptible and resistant ceftolozane-tazobactam profiles were subsequently analyzed by whole genome sequencing (WGS) (Table S1) [16].

### 2.2. Sequence Analysis

WGS was performed using the Illumina-Hiseq 4000/NovaSeq 6000 platforms with 2 × 150 pb paired-end reads (Oxford Genomics Center, Oxford, UK). FastQC (v0.11.8) (https://www.bioinformatics.babraham.ac.uk/projects/fastqc/ (accessed on 10 February 2021)) and Prinseq-lite-0.20.3 (http://prinseq.sourceforge.net/ (accessed on 10 February 2021)) tools were used for quality control and sequences filtering, respectively [17]. Sequence reads were de novo assembled using SPAdes (v3.11.1) and assembled contigs were evaluated by QUAST (v5.0.2) [18,19]. Bacterial identification was confirmed by the Taxonomic Sequence Classification System Kraken (v1.0) [20]. Draft genomes were annotated by Prokka (v1.13.3) [21].

### 2.3. Ciprofloxacin Resistance Determinants

Acquired quinolone resistance genes were screened using Abricate (v0.8.11) (ARG-ANNOT and ResFinder databases; threshold, 95% identity; 90% coverage). In addition, mutations from 34 chromosomal genes involved in fluoroquinolones resistance were also analyzed using SNIPPY software (v4.4.3) (https://github.com/tseemann/snippy (accessed on 10 February 2021)) (Table S2) [16]. Briefly, all *P. aeruginosa* assembled sequences were mapped against the *P. aeruginosa* PAO1 reference genome (GenBank accession No. NC_002516.2) using Snippy/BWA-MEM (v0.7.17) tool. Variant calling was performed using Snippy/Freebayes (v1.3.1) software (minimum base quality of 20, minimum read coverage of 10X and 90% read concordance at a locus). Single nucleotide polymorphisms (SNPs) and small insertions and deletions (InDels) annotation were carried out by Snippy/SnpEff (v4.3) software (http://snpeff.sourceforge.net/index.html (accessed on 10 February 2021)). Synonymous SNPs and SNPs distributed among all *P. aeruginosa* isolates were not considered to be correlated to ciprofloxacin resistance phenotypes. Frameshift mutations and premature stop codons were considered to result in the inactivation of the corresponding protein.

The presence of *crpP*-*like* genes was confirmed using Blastn tool (v2.9.0+) (http://blast.ncbi.nlm.nih.gov/Blast.cgi (accessed on 10 February 2021)) and the reference gene NG_062203.1. CrpP proteins alignment was performed using the Clustal Omega tool (https://www.ebi.ac.uk/Tools/msa/clustalo/ (accessed on 10 February 2021)).

### 2.4. Genetic Context of crpP Gene

*CrpP*-carrying *P. aeruginosa* genomic islands (PAGI) were reconstructed, and a comparative map was drawn using Blast, SnapGene and Kablammo tools (http://kablammo.wasmuthlab.org/ (accessed on 10 February 2021)) [22]. pUM505 plasmid sequence (HM560971) and PAGIs’ sequences with accession numbers MT074669, MT074670, MT074671, MT074672, MT074673, MT074674 and MT074675 were used as reference.

### 2.5. Statistical Analysis

Fisher’s exact test was used to analyze associations between categorical variables. Statistical analysis was performed using R software (RStudio Team 2016 v1.0.44, RStudio, Boston, MA, USA). A *p*-value < 0.05 was considered as statistically significant.

### 2.6. Accession Numbers

PAGIs’ sequences were deposited in the GenBank database under the following accession numbers: PAGI-*crpP1.1* (MT577544), PAGI-*crpP1.2* (MT577545), PAGI-*crpP1.3* (MT577546), PAGI-*crpP2* (MT577547), PAGI-*crpP3* (MT577548), PAGI-*crpP4.1* (MT577549), PAGI-*crpP4.2* (MT577550), PAGI-*crpP4.3* (MT577551), PAGI-*crpP5* (MT577552) and PAGI-*crpP6* (MT577553). CrpP5- and CrpP6-encoding genes accession numbers are MT544449 and MT577543, respectively.

## 3. Results

### 3.1. Ciprofloxacin Resistance in STEP and SUPERIOR P. aeruginosa Isolates

According to our previous studies, during the STEP (Portugal) and SUPERIOR (Spain) surveillance studies, the ciprofloxacin resistance rate among the MDR/XDR *P. aeruginosa* isolates was 56% (222/396) and 55% (44/80), respectively. Interestingly, almost all *P. aeruginosa* isolates that displayed non-susceptibility to ceftolozane-tazobactam (MIC_CT_ > 4 mg/L) (STEP 5.3% (21/396) and SUPERIOR 8.7% (7/80)) showed also a ciprofloxacin resistant phenotype (MIC_CIP_ > 2 mg/L) (STEP 90.5% (19/21), SUPERIOR 85.7% (6/7)), and a statistical co-relation was established (STEP, *p* < 0.001; SUPERIOR, *p* = 0.006) (Table 1) [14,15].

### 3.2. P. aeruginosa Genome Analysis and Molecular Typing

A total of 55 MDR/XDR-*P. aeruginosa* isolates from 11 Portuguese and 8 Spanish hospitals were analyzed by WGS as a part of the STEP and SUPERIOR studies, respectively [16]. In this subset, 46 isolates (83.6%) showed a non-wild type ciprofloxacin susceptible phenotype (MIC_CIP_ = 1 –> 2 mg/L) (Table 2, Table S2). In agreement with our previous study, a total of 16 different clonal complex (CC) were detected. Moreover, the clones CC235 (15/15), CC175 (9/10), CC348 (5/5) and CC244 (4/7) were predominant among the ciprofloxacin resistance strains (Table S2).

### 3.3. Acquired Resistance Genes

PMQR genes were detected in 6 *P. aeruginosa* isolates (10.9%). *qnrS*2 gene was identified in two clonally related *P. aeruginosa* strains belonging to the CC309 from the same Spanish center. Moreover, both CC309 strains and four other isolates belonging to the CC348 and recovered in the same Portuguese hospital carried the *aac*(*6′)-Ib-cr* gene. All these isolates displayed a ciprofloxacin resistance phenotype (MIC_CIP_ > 2 mg/L), although statistical significance was not observed (Table 2, Table S2).

### 3.4. Mutational Resistome

Single nucleotide polymorphisms (SNPs) and small insertions and deletions (InDels) were found in 91.2% (31/34) of the chromosomal genes known to be involved in fluoroquinolone resistance (Table S2).

Mutations associated with the QRDR were detected in 85.5% (47/55) of isolates and were statistically related to the ciprofloxacin resistance phenotype (*p* = 0.02) (Table 2, Table S2). Note that the previously described GyrA-T83I, GyrA-D87N, ParC-S87W and ParC-S87L substitutions were found in 36 (65.4%), 9 (16.4%), 10 (18.2%) and 21 (38.2%) isolates, respectively. Interestingly, except in one case, mutations in *gyrA* and *parC* genes were not detected in ciprofloxacin susceptible strains, and a statistical correlation was demonstrated (*p* = 0.03 and *p* = 0.01, respectively) (Table 2, Table S2).

Mutations in efflux pumps regulatory genes such as *nalC* and *nfxB* were also detected in 87.3% (48/55) and 7.3% (4/55) of the *P. aeruginosa* isolates, but a statistically significant correlation with a decreased ciprofloxacin susceptibility was not observed (Table 2).

### 3.5. crpP-Encoding Genes

**Figure 1:**
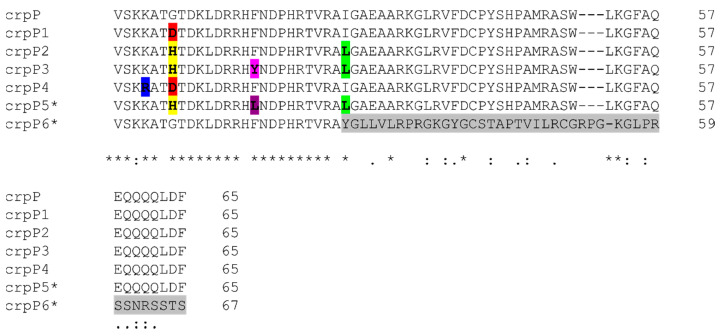
Alignment of the amino acid sequences of CrpP variants detected in *P. aeruginosa* isolates recovered during the STEP (Portugal) and SUPERIOR (Spain) surveillance studies (CrpP1, WP_023102333.1; CrpP2, WP_155687830.1; CrpP3, WP_155684864.1; CrpP4, WP_152934806.1; CrpP5, MT544449; CrpP6, MT577543). CrpP (NG_062203.1) was used as reference. Bold letters indicate the mutations detected in CrpP proteins: K4R (blue), G7D (red), G7H (yellow), F16Y (pink), F16L (purple), I26L (green) and I26fs (gray). * crpP5 and crpP6 variants were described during this study. Fs = frameshift mutation.

Ten different *crpP*-encoding genes were found in 36 (65.4%) isolates (25 from Portugal and 11 from Spain) resulting in six CrpP enzymes named CrpP1, CrpP2, CrpP3, CrpP4, CrpP5 and CrpP6 (Table 3, Figure 1). *crpP5-* (G7H, F16L, I26L; accession number MT544449) and *crpP6*-encoding genes (I26fs; accession number MT577543) were first described in this study.

The overall prevalence of *crpP* genes in MDR/XDR *P. aeruginosa* isolates was 59. 5% (25/42) and 84.6% (11/13) in Portugal and Spain, respectively. Moreover, *crpP-like* genes were found in a wide variety of *P. aeruginosa* clones (13/16) in both countries (11/14 in Portugal and 2/3 in Spain) (Table S2). CrpP1 (G7D), CrpP2 (G7H, I26L), CrpP3 (G7H, F16Y, I26L) and CrpP4 (K4R, G7D) variants were the most represented in our collection (CrpP1 (23/36), CrpP2 (4/36), CrpP3 (4/36) and CrpP4 (3/36) and were mostly linked to certain clones (Table 3). CrpP1 was the predominant variant and was identified among non-related clones of both countries including the CC175 (Spain) high-risk clone and the CC348 (Portugal) clone (Table 3, Table S2). Note that *crpP*-*like* gene was only found in one *P. aeruginosa* isolate belonging to the CC235 (1/15), the most frequent clone during this study (Table S2).

Correlation between the detection of *crpP* genes and the resistance to ciprofloxacin could not be precisely established (Table 2). In fact, 8 of 9 ciprofloxacin susceptible isolates (MIC_CIP_ = 0.25–0.5 mg/L) contained a CrpP protein (CrpP2 (*n* = 3), CrpP1 (*n* = 2), CrpP4 (*n* = 2) and CrpP5 (*n* = 1)). Furthermore, CrpP2 variant was detected in CC244 isolates in which QRDR mutations were not found and statistical significance could be established with the ciprofloxacin susceptible phenotype (Table 2). Interestingly, with the exception of CrpP6, all CrpP1-crpP5 variants carried a missense mutation in the G7 amino acid position, essential to the function of the protein, and a correlation with a decreased susceptibility to ciprofloxacin was not determined (Table 3) (Figure 1). Additionally, mutations in the I26 amino acid position were also found in 3 of 6 variants (CrpP2, CrpP3 and CrpP5) (Figure 1). Note that the CrpP6 producing *P. aeruginosa* isolate (CC449, center 2) showed a ciprofloxacin resistant phenotype (MIC_CIP_ = 1 mg/L), but mutations in QRDR or the presence other acquired resistance genes related to decreased susceptibility to fluoroquinolones were not found (Table S2).

### 3.6. crpP-Carrying Pathogenicity Genomic Islands (PAGI)

**Figure 2:**
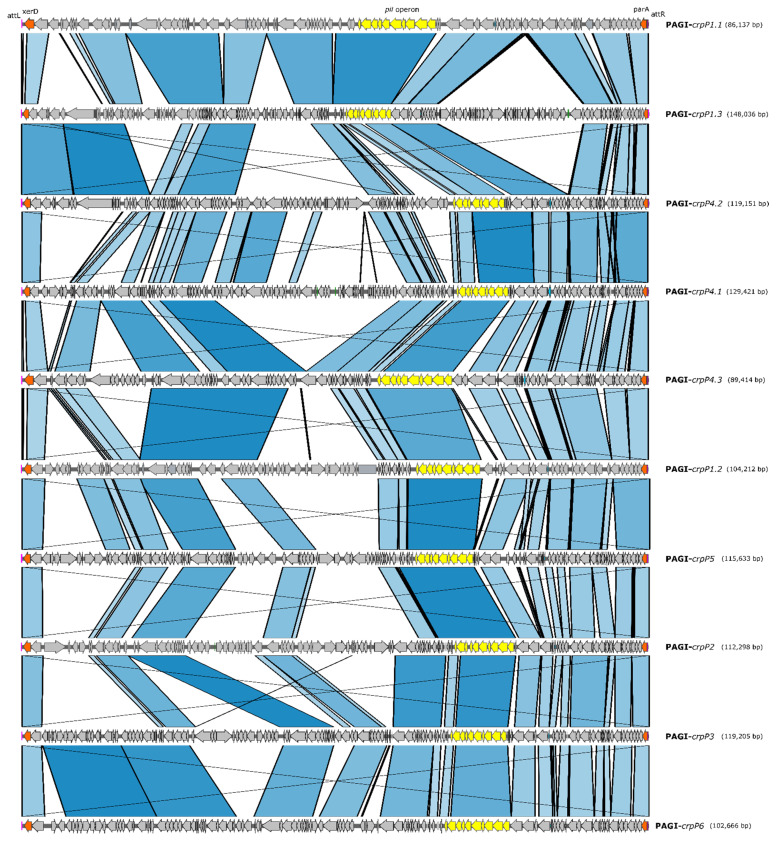
Map of sequence comparison of *crpP-like*-carrying PAGI reconstructed from *P. aeruginosa* isolates recovered during the STEP (Portugal) and SUPERIOR (Spain) surveillance studies. Arrows represent genes and indicate the orientation. Relevant genes are indicated by colored arrows as follows: attL and attR sequences (pink), *xerD* and *parA* genes (orange), *pil* operon (yellow) and *crpP-like* genes (blue). Blue bands indicate the portions of the PAGI sequences that align to each other.

WGS analysis confirmed that all *crpP*-*like* genes were located in a large chromosomal region flanked by *attL* and *attR* sequences. *xer*D and *par*A genes involved in the mobilization and integration of pathogenicity genomic islands (PAGI) structures were also identified flanking this region. Ten PAGIs were reconstructed using one representative isolate harboring a different *crpP* gene (Figure 2). Our *crpP*-carrying *P. aeruginosa* strains contained related PAGIs varying in size (ca. 85–150 Kb). Moreover, common regions involved in mobilization and maintenance such as the *pil* operon were found in all PAGIs (Figure 2).

PAGI-*crpP1.1* (ca. 85 Kb) was the most frequent genomic island (20/55) and was found in different clones in 5 centers from Portugal (CC348, CC253, CC308 and CC179) and 5 centers from Spain (CC175) (Table 2, Table S2).

## 4. Discussion

In the last thirty years, quinolones have been widely used to treat infections caused by both Gram-positive and Gram-negative bacteria in different infection sites. However, the high level of use of these antimicrobials has contributed to the parallel selection and dispersion of quinolone-resistant pathogens in both hospital and community settings [6]. During the STEP (Portugal) and SUPERIOR (Spain) surveillance studies, that monitor ceftolozane-tazobactam susceptibility, 55–56% of MDR/XDR *P. aeruginosa* clinical isolates recovered from ICU patients showed a ciprofloxacin resistant phenotype (MIC_CIP_ > 2 mg/L). Moreover, a statistical association was found between the ciprofloxacin resistance and the resistant ceftolozane-tazobactam phenotype in both STEP and SUPERIOR collections. Ceftolozane-tazobactam is a new cephalosporin-β-lactamase inhibitor combination with a wide antimicrobial spectrum and high activity against MDR Gram-negative bacteria, with the exception of carbapenemase producers [23]. However, ceftolozane-tazobactam resistance has been recently described in ESBL-producing *Enterobacterales* populations, including those recovered during the STEP and SUPERIOR surveillance studies [24,25]. The selection pressure in the intestinal microbiota caused by the extensive use of antibiotics during decades has led to the emergence of MDR/XDR pathogens carrying multiple antibiotic resistance determinants, including mechanisms involved in quinolone resistance but also β-lactamases genes such as ESBL and carbapenemases.

In consistence with previous studies, the most frequent resistance mechanism to quinolones in our MDR/XDR *P. aeruginosa* sequenced isolates was the presence of mutations in the *gyrA* and *parC* genes, encoding gyrase and topoisomerase IV subunits [4]. Additionally, the horizontally transferred genes *qnrS2* and *aac*(*6′)-Ib-cr* were eventually detected in relation to certain clones. On the other hand, ten different *crpP*-encoding genes resulting in six CrpP enzymes were also found in 65.4% of MDR/XDR *P. aeruginosa* clinical isolates. Four of these CrpP variants (CrpP1, CrpP2, CrpP3 and CrpP4) have been previously found in *P. aeruginosa* isolates from other European countries [11], but two novel CrpP proteins (CrpP5 and CrpP6) were first identified in our study in two Portuguese centers.

*CrpP*-*like* genes have been recently described as a novel resistance mechanism affecting ciprofloxacin in *P. aeruginosa* and other MDR Gram-negative bacteria [9,13]. However, in our *P. aeruginosa* collection, decreased susceptibility to ciprofloxacin was not correlated to the presence of *crpP* genes. In fact, in the absence of mutations in QRDR or other PMQR genes such as *qnrS2* and *aac(6′)-Ib-cr*, only the novel *crpP6*-encoding gene could be associated with a decreased ciprofloxacin susceptibility. It should be noted that the remaining CrpP variants (CrpP1-CrpP5) carried a missense mutation in the G7 amino acid, previously defined in a recent study as an essential residue for *crpP*-mediated ciprofloxacin resistance [12]. G7 amino acid (involved in catalysis) and I26 amino acid (involved in ATP binding) are located at the N-terminal region and are suggested to be conserved residues essential for the function of CrpP protein [12]. According to recent literature, mutations in G7 and I26 position are non-commonly detected among the amino acid sequences of CrpP present in GenBank [6,26]. However, in both Portuguese and Spanish collections, CrpP variants carrying mutations in G7 were predominant, as recently described in other European countries [11]. It should be noted that the emergence and dissemination of modified CrpP variants in *P. aeruginosa* clinical isolates circulating in nosocomial settings such as ICUs could lead to the appearance and dissemination of novel *crpP* alleles with a higher ability to affect quinolones.

Interestingly, all *crpP*-encoding genes were located in PAGIs closely related to other genomic islands previously found in Europe between 2000 and 2015 [11]. Previous studies have demonstrated that *xerD* and *parA* genes contribute to transfer PAGI through *P. aeruginosa* strains, contributing to the dissemination of different antibiotic resistance mechanisms and virulence determinants and leading to the evolution and expansion of this species among different habitats [27]. Additionally, homologues of *crpP*-*like* gene have also been identified in other Gram-negative bacteria such as ESBL producing *Enterobacterales* [13]. The spread of *crpP*-*like* genes among different species and clones through well-conserved genetic platforms could be also a consequence of the selective pressure caused by the extensive use of ciprofloxacin in the treatment of a large number of infections.

According to our results, PAGI-*crpP1.1* was the most widely disseminated genomic island. Furthermore, the dissemination of this conserved PAGI-*crpP1.1* occurred among non-clonally related isolates distributed in both countries, including clones broadly disseminated in our region such as CC175 high-risk clone (Spain) and CC348 clone (Portugal). Note that prevalence of *crpP* genes was higher in the *P. aeruginosa* isolates recovered in Spain than in those recovered in Portuguese hospitals, and this finding could be related to a successful association between the PAGI-*crpP1.1* and the CC175 high-risk clone. It should be noted that the CC175 is the most frequent *P. aeruginosa* clone in Spanish hospitals and that its extensive resistome has been found to be determined mainly by mutational events [16,28,29]. On the other hand, the presence of *crpP* genes was scarcely observed in the CC235 (1/15), the most frequent clone in the Portuguese collection. However, CC235-*P. aeruginosa* harboring homologues of *crpP* genes has been reported as the most prevalent clone among clinical isolates recovered in France and Switzerland between 2000 to 2015 [11]. In this sense, the presence of chromosomal *crpP*-*like* genes with a high capacity of mutation in well-adapted hospital *P. aeruginosa* lineages such as CC175 and CC235 could contribute to the emergence of novel *crpP* genes with a higher spectrum of activity against fluoroquinolones.

On the other hand, genomic islands of *P. aeruginosa* have previously been shown to carry different virulence factors generating hypervirulent strains with a high adaptative capacity [30]. Fortunately, although our collection of *P. aeruginosa* carried a high virulence gene content, virulence factors were not detected in the *crpP*-*like*-carrying PAGIs [16].

According to our results, mutations in gyrase and topoisomerase encoding genes, particularly in *gyrA* and *parC*, are the main resistance mechanism to ciprofloxacin in MDR/XDR *P. aeruginosa* clinical isolates from ICU patients in Portugal and Spain. Additionally, transferable mechanisms of quinolone resistance such as *qnrS2* and *aac*(*6′)-Ib-cr* genes were also detected in a low proportion of strains. Nevertheless, the spread of different *crpP*-*like* genes among non-related *P. aeruginosa* clones through conserved and wide genomic islands may contribute to the emergence and persistence of MDR/XDR pathogens in hospital settings and should be further explored.

## 5. Conclusions

*CrpP*-encoding genes are frequently detected in the chromosome of non-related clones of *P. aeruginosa* isolates causing infections in ICU patients from Portugal and Spain. Moreover, *crpP*-*like* genes were located in PAGI structures with conserved regions involved in mobilization and integration and related to genomic islands previously found in *P. aeruginosa* clinical isolates recovered in other European countries. In our collection, inactive or unfunctional CrpP proteins were predominant, and ciprofloxacin resistance was more probably mediated by mutations in the QRDR or eventually due to the presence of horizontally transferred genes other than *crpP*. Nevertheless, highly mobilizable PAGIs harboring different homologous *crpP* genes with a high capacity of mutation could play a critical role in the dissemination and transmission of other antimicrobial resistance genes among nosocomial *P. aeruginosa* high-risk clones in Europe and should be considered for further studies.

## Figures and Tables

**Table 1:** Ciprofloxacin resistance rates in ceftolozane-tazobactam resistant and susceptible *P. aeruginosa* clinical isolates detected during the STEP (Portugal) and SUPERIOR (Spain) surveillance studies.

		CT-R		CT-S		*p*-Value	Odd Ratio (95%CI)
	Total No.	CIP-R No. (%)	CIP-S No. (%)	CIP-R No. (%)	CIP-S No. (%)		
**STEP**	396	19 (90.5)	2 (9.5)	128 (34.1)	247 (65.9)	<0.001	18.2 (4.3–163.5)
**SUPERIOR**	80	6 (85.7)	1 (14.3)	22 (30.1)	51 (69.9)	0.006	13.4 (1.5–649.5)

CT = ceftolozane-tazobactam; CIP = ciprofloxacin. Ceftolozane-tazobactam EUCAST-2020 breakpoint [susceptible (S) ≤ 4 mg/L; resistant (R) > 4 mg/L]; Ciprofloxacin EUCAST-2020 breakpoint (susceptible (S) ≤ 0.001 mg/L; susceptible, increased exposure (I) > 0.001–0.5 mg/L; resistant (R) > 0.5 mg/L).

**Table 2:** Percentage of susceptible, increased exposure (I) and ciprofloxacin resistant (R) *P. aeruginosa* clinical isolates detected during the STEP and SUPERIOR surveillance studies and fluoroquinolone resistance mechanisms involved.

	Total (*n* = 55)	CIP ^a^ Susceptibility Profile *		*p*-Value	Odd Ratio (95%CI)
		I [No. (%)] (*n* = 9)	R [No. (%)] (*n* = 46)		
**ARG ^b^**	**6 (10.9)**	**0**	**6 (13)**	**0.57**	**0 (0–4.55)**
*qnrS2*	2 (3.6)	0	2 (4.3)	0.57	0 (0–4.55)
*aac(6′)-Ib-cr*	6 (10.9)	0	6 (13)	1	0 (0–28.23)
**QRDR ^c^**	**47 (85.5)**	**5 (55.6)**	**42 (91.3)**	**0.02**	**7.90 (1.12–59.46)**
GyrA	42 (76.4)	4 (44.4)	38 (82.6)	0.03	5.69 (0.99–35.95)
GyrB	1 (1.8)	0	1 (2.2)	NA	NA
ParC	40 (72.7)	3 (33.3)	37 (80.4)	0.01	7.81 (1.37–57.90)
ParE	26 (47.3)	4 (44.4)	22 (47.8)	1	1.14 (0.21–6.54)
**CrpP ^d^**	**36 (65.5)**	**8 (88.9)**	**28 (60.9)**	**0.14**	**0.20 (0.01–1.70)**
CrpP1	23 (41.8)	2 (22.2)	21 (45.6)	0.27	2.89 (0.48–31.41)
CrpP2	4 (7.2)	3 (33.3)	1 (2.2)	0.01	0.05 (0.001–0.72)
CrpP3	4 (7.2)	0	4 (8.7)	NA	NA
CrpP4	3 (5.4)	1 (11.1)	2 (4.3)	0.07	11.89 (0.55–769.73)
CrpP5	1 (1.8)	1 (11.1)	0	NA	NA
CrpP6	1 (1.8)	0	1 (2.2)	NA	NA
***nalC*^e^**	**48 (87.3)**	6 (66.7)	42 (91.3)	0.08	0.20 (0.03–1.70)
***nfxB*^e^**	**4 (7.3)**	2 (22.2)	2 (4.3)	0.12	5.97 (0.38–95.35)

^a^ CIP = Ciprofloxacin; ^b^ ARG = acquired resistance genes; ^c^ QRDR = quinolone resistance-determining region; ^d^ Presence of genes/proteins; ^e^ Mutated proteins; * Ciprofloxacin EUCAST-2020 breakpoint (susceptible (S) ≤ 0.001 mg/L; susceptible, increased exposure (I) > 0.001–0.5 mg/L; resistant (R) > 0.5 mg/L).

**Table 3:** CrpP enzymes detected in the *P. aeruginosa* isolates from STEP and SUPERIOR surveillance studies.

Enzyme (No. of Isolates)	Gene (No. of Isolates)	Mutations (nt) *	Missense Mutations (aa) *	Clonal Complex (No. of Isolates)	CIP-MIC Interpretation (No. of Isolates)
CrpP1 (23)	*crpP1.1* (20)	20(A-G), 192(C-T)	G7D	CC175 (10), CC348 (5), CC253 (3), CC179 (1), CC308 (1)	I (2), R (18)
	*crpP1*.2 (2)	20(A-G), 66(C-T)		CC554 (2)	R (2)
	*crpP1*.3 (1)	1 (T-C), 20(A-G), 192(C-T)		CC235 (1)	R (1)
CrpP2 (4)	*crpP2* (4)	19(CA-GG), 39(G-A), 45(T-C), 76(C-A), 81(C-T), 123(T-C), 183(A-G), 192(C-T)	G7H, I26L	CC244 (4)	I (3), R (1)
CrpP3 (4)	*crpP3* (4)	19(CA-GG), 39(G-A), 45(T-C), 47(A-T), 76(C-A), 81(C-T), 123(T-C), 183(A-G), 192(C-T)	G7H, F16Y, I26L	CC244 (3), CC313 (1)	I (1), R (3)
CrpP4 (3)	*crpP4*.1 (1)	11(A-G), 20(A-G), 138(A-G)	K4R, G7D	CC27 (1)	I (1)
	*crpP4*.2 (1)	11(A-G), 20(A-G), 192(C-T)		CC446 (1)	R (1)
	*crpP4*.3 (1)	11(A-G), 18(T-C), 20(A-G), 174(A-G), 183 (A-G)		CC179 (1)	R (1)
CrpP5 (1)	*crpP5* (1)	19(CA-GG), 39(G-A), 45(CT-TC), 76(C-A), 81(C-T), 123(T-C), 162(A-G), 183(A-G), 192(C-T)	G7H, F16L, I26L	CC971 (1)	I (1)
CrpP6 (1)	*crpP6* (1)	33(A-G), 73(insGCTTACGG)	I26fs	CC499 (1)	R (1)

CC = clonal complex; fs = frameshift mutation; CIP = ciprofloxacin; I = susceptible, increased exposure (MIC > 0.001–0.5 mg/L); R = resistance (MIC > 0.5 mg/L). * SNPs and InDels mutations were obtained using the crpP reference gene NG_062203.1.

## Data Availability

The sequence data analyzed in this study are openly available at NCBI [see Material and Methods section for Accession numbers].

## Supplementary Materials

The following are available online at https://www.mdpi.com/2076-2607/9/2/388/s1, Table S1: MDR/XDR-*P. aeruginosa* clinical isolates analyzed by whole genome sequencing during the SUPERIOR (Spain, 2016–2017) and STEP (Portugal, 2017–2018) surveillance studies; Table S2: Chromosomal genes detected in *P. aeruginosa* isolates as a part of the mutational resistome affecting fluoroquinolones susceptibility. *crpP* gene content, epidemiological data, acquired antibiotic resistance genes and ciprofloxacin-susceptibility results are also included.
